# Functional Nutrients and Jujube-Based Processed Products in *Ziziphus jujuba*

**DOI:** 10.3390/molecules29143437

**Published:** 2024-07-22

**Authors:** Weitong Cai, Haining Zhuang, Xiaoyu Wang, Xia Fu, Sheng Chen, Lingyun Yao, Min Sun, Huatian Wang, Chuang Yu, Tao Feng

**Affiliations:** 1 School of Perfume and Aroma Technology, Shanghai Institute of Technology, Shanghai 201418, China; caiweitong830@163.com (W.C.); wanghuatian@sit.edu.cn (H.W.);; 2 School of Health and Society Care, Shanghai Urban Construction Vocational College, Shanghai 201100, China; 3 Hunan Wuzizui Industrial Group Co., Ltd., Xiangtan 411228, China

**Keywords:** jujube, nutrients, processed jujube products, bioactive compounds, pharmacological activity

## Abstract

Jujube (*Ziziphus jujuba* Mill.) is the first tree species in China, with a long history and abundant yield. However, fresh jujubes have a short shelf-life and are not resistant to storage. Therefore, more and more processed jujube products are being studied. These processed products can extend the shelf-life of jujubes and attract widespread attention for their rich functional nutrients. This review summarized changes in nutrients of fresh jujube and processed products and the research progress of different preparation methods of jujubes. Meanwhile, the pharmacological effects of bioactive components in jujube-based products were concluded. Jujube and its processed products contain rich polysaccharides, vitamin C, and other functional nutrients, which are beneficial to humans. As the initial processing method for jujubes, vacuum freezing or microwave drying have become the most commonly used and efficient drying methods. Additionally, processed jujube products cannot be separated from the maximum retention of nutrients and innovation of flavor. Fermentation is the main deep-processing method with broad development potential. In the future, chemical components and toxicological evaluation need to be combined with research to bring consumers higher quality functional jujube products and ensure the sustainable development of the jujube industry.

## 1. Introduction

Jujube (*Ziziphus jujuba* Mill.) is a plant in the *Rhamnaceae* family, native to China. It was harvested and consumed directly by people as early as the Neolithic era over 7000 years ago [1]. Subsequently, due to the large quantity of jujube fruit, it became a wild fruit tree, and, in the Han Dynasty, there began to be a certain scale of jujube planting areas, and jujube began to be introduced to the countries around China and then to Central Asia and Europe [2]. The ancients of the “Erya” recorded eleven kinds of jujube, which shows that jujube cultivation has a long history in China, and also lays the foundation for extensive planting areas and diverse varieties of Chinese jujube. Nowadays, the total area of Chinese jujube trees exceeds 2 million hectares, accounting for about 98% of the global jujube germplasm resources. The annual output of exported jujube fruits is as high as 10,000 tons or more [3,4].

Jujube is a homology of medicine and food with high nutritional value, rich in carbohydrates, proteins, dietary fiber, and vitamins. Fresh jujube contains 200–800 mg/100 g of vitamin C, and is known as the natural “Vitamin C pill” [2,5]. It also contains rich fatty acids and various amino acids, including 8 amino acids essential for the human body [6]. In addition, bioactive components such as polysaccharides, triterpenoids, etc., are also important sources of jujube, which has health benefits such as anti-obesity, anti-cancer, and antioxidant effects [7,8].

**Figure 1:**
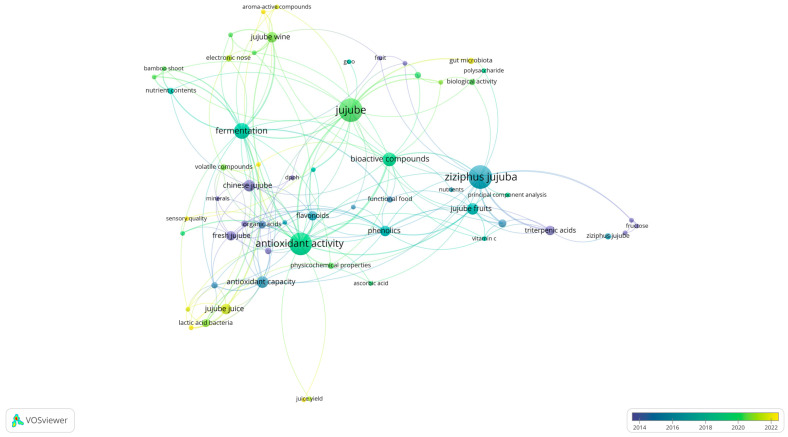
The literature search on the nutritional components of jujubes and jujube-based products in the past 15 years (drawn through VOSviewer 1.6.20 software).

However, as a unique Chinese fruit resource, fresh jujubes have a relatively short storage time after picking. In order to extend the shelf-life of jujubes for sale, the production methods were mainly preliminary processing, like in the form of dried jujube [8]. As consumers pay more attention to nutrition and health, more and more functional jujube products and new types of jujube snacks are being launched on the market. Based on this, this paper organized the articles on nutrients in different varieties of jujube, the research progress, and pharmacological effects of jujube and its processed products in the past 15 years, proposing countermeasures and reference values for the sustainable development and higher quality development of the jujube industry. Figure 1 shows the co-occurrence of keywords related to jujube research in the past 15 years.

## 2. Current Status of Jujube Resources

According to Li Y et al., jujube initially originated in northern China in the lower reaches of the Yellow River in the Qihe region [9]. In ancient times, wild jujube fruit trees were extremely common in this area, making it a target for consumption and cultivation by people [10]. Comparing carbonized jujube fruits from the Qihe River Basin site area with modern jujube fruit specimens, it was speculated that ancient people increased jujube pulp production in order to meet food needs [9]. Nowadays, most of the jujube morphology can be found in the wild jujube [1], and provides strong evidence of the domestication of the wild jujube trees.

With the continuous cultivation by people, the planting range of jujube trees has gradually expanded. As of the year 2022, there are about 950 kinds of recorded and preserved jujube varieties in China, and most of them that have been distributed in Europe, Southeast Asia, and the United States were imported from China [11]. The mainly exported jujube varieties include: *Ziziphus jujuba* cv. Jinsixiaozao, *Z. jujuba* cv. Huizao, *Z. jujuba* cv. Junzao, *Z. jujuba* cv. Dongzao, etc., and Table 1 shows 10 famous jujube varieties in China. With the climate and quality differences of jujube, Qu & Wang divided Chinese jujube resources into two regions, as the Qinglin Huaihe benchmark for the north and south regions [10]. The northern jujube north of the Qinling and Huaihe Rivers has long sunshine time and high sugar content and is often sun-dried and processed into dried jujube, while the southern one grows in humid and acid-loving areas, with larger fruits but low sugar content, mainly used to make jujube honey [4]. Table 1 provides a detailed summary of different jujube trees [4,10].

## 3. Characteristics of Nutritional Components in Jujubes

Jujubes have high nutritional value due to their high content of nutrients and non-volatile chemical compounds [12,13]. Therefore, we classified the nutritional components of jujubes into three categories for a more comprehensive discussion. Table 2 lists a comparison between nutrients in fresh and dried jujube in detail.

### 3.1. Macronutrients

Overall, macronutrients are nutrients that the human body needs to consume in large quantities, and jujube is rich in macronutrients [8]. In fresh jujube, carbohydrate content ranges from 23–32% and protein content is 1.2%, while when fresh jujube is processed and dried, carbohydrate and protein content change to 63–76.3% and 2.9–6.3%, respectively [2]. Gao et al. compared the sugar content of fresh jujube with dried and found that the glucose and fructose content increased significantly after drying [14]. Therefore, glucose and fructose are the main soluble sugars in jujube.

Dietary fiber is also a part of carbohydrates. Although it may affect the absorption of other nutrients such as vitamin C, dietary fiber can control excessive calorie intake. Li et al. determined the nutritional components of dried Chinese jujubes and concluded that the fiber content ranged from 6.03% to 8.90% [15].

Amino acids are important compounds in protein synthesis and flavor components. Previous studies have shown that different jujubes contain 17–29 types of free amino acids, which include 8 essential amino acids that cannot be synthesized on their own [16].

Li et al. determined 5 famous Chinese varieties, and the results showed that the carbohydrate, protein, and fat content was 80.86–85.63%, 4.75–6.86%, and 0.37–1.02%, respectively [15]. In addition, Pareek S summarized that in Indian varieties of jujubes, fresh jujubes contained 81–83% water and 17.0% carbohydrates [17], and Choi et al. compared two Korean jujube varieties and showed that the water content in fresh jujube was 71.46–72.9%, the protein content was 1.37–1.71%, and the fat content was 0.31–0.33% [18].

Hernández et al. examined four Spanish jujubes and found that the brix value ranged from 14.6 to 18.4% [12]. Afterwards, the team also examined the nutritional composition of three types of Spanish jujube and found a more precise range of fructose and sucrose content, with 6.76–8.84% and 7.3–9.51%, respectively [19]. Xinjiang has a wide range of planting areas, therefore Chen et al. compared jujubes from eight Xinjiang production areas and found that fresh jujubes had 1.87–3.97% protein and 27.19–31.7% total sugar [20]. These studies confirm that jujubes contain a large number of macronutrients, the content of which varies depending on jujube variety, origin, and testing method.

### 3.2. Micronutrients

Micronutrients refers to vitamins and minerals, which are crucial for the normal functioning of the body. Previous research on jujubes has shown that they have high nutritional value, are rich in vitamins A, B, C and their complexes, major elements such as potassium (K), magnesium (Mg), calcium (Ca), phosphate (P), and trace elements such as iron (Fe), manganese (Mn), copper (Cu), zinc (Zn) [18]. Li et al. found that K, P, Ca, and Mn were the main mineral components of jujubes, followed by Na, Fe, Zn, and Cu, while Yazao has the highest K content at 458 mg/100 g and Jinsizao has the highest P content at 110 mg/100 g. At the same time, it was also found that jujubes contain a large amount of vitamin C (192–359 mg/100 g) [15].

Selenium-containing fertilizers began to be applied in fruit tree cultivation. Selenium (Se) has an important role as a trace element in plant growth and human health. Wu et al. applied exogenous Se to jujube trees. It was found that exogenous Se application could increase the vitamin C content in jujube fruits (337–496 mg/100 g) [21]. In addition, in four Spanish jujubes, K had the highest content, at 1190–1730 mg/100 g, followed by Mg and Ca elements, and vitamin C, with a content of 410–640 mg/100 g [12]. In 2021, a study found that the vitamin C content of 11 fresh Indian jujubes ranged from 55.27 mg/100 g to 164.47 mg/100 g [22]. It can be seen that the growth environment has a certain impact on the quality of jujubes.

### 3.3. Bioactive Components

Bioactive compounds have specific physiological functions in living organisms and can affect their growth and metabolism. Jujubes contain polysaccharides, triterpenoids, flavonoids, polyphenolic compounds, and other substances that are considered potential sources of nutritional application and key factors determining the quality of jujubes [23]. Kou et al. evaluated the nutritional composition of 15 Chinese jujubes and determined that the total polyphenolic compound content was 0.558–2.520 mg GAE/g FW, total flavonoid content was 0.47–2.0 mg RE/g FW, jujube polysaccharide content was 3.103–21.815 mg/g FW, and cAMP content was 17.38–193.93 ug/g FW [24]. As a more effective natural antioxidant, jujube had a higher total phenolic compound content than cherry (114.6 mg GAE/100 g FW), guava (194.1 mg GAE/100 g FW), and apple (272.1 mg GAE/100 g FW) [2].

**Table 2:** Detailed contents of every nutrient in fresh or dried jujube.

Varieties of Jujube	Nutrients Name (Unit)	Contents		Reference
		Fresh Jujube	Dried Jujube	
Summary of Chinese jujubes	Moisture (%, *w*/*w*)	73.4	14–27.8	[2]
	Protein (%, *w*/*w*)	1.2	2.9–6.3	
	Lipid (%, *w*/*w*)	0.2	0.3–2.3	
	Dietary fiber (%, *w*/*w*)	1.6	1.8–3.1	
	Vitamin C (mg/100 g)	200–800	12–29	
	Calcium (g/kg)	14	20–63	
	Phosphorus (mg/100 g)	23	55–75	
	Iron (mg/100 g)	0.5	1.6–3.1	
	cAMP (μg/g)	-	40–400	
	Total sugar (%, *w*/*w*)	23–32	63–76.3	
	Carotene (mg/100 g)	0.01	0.01	
	Thiamin (mg/100 g)	0.06	0.06	
	Lactoflavin (mg/100 g)	0.04	0.3	
	Niacin (mg/100 g)	0.6	1.2	
*Z. jujuba* cv. Muzao (Chinese jujube)	Total flavonoids (mg RE/100 g FW)		62.0–284.9	[14]
	Total polyphenol (mg GAE/100 g FW)		275.6–541.8	
	Proanthocyanidins (mg CE/100 g FW)		58.0–413.7	
	Ascorbic acid (mg/100 g)		225.1–387.9	
*Zizipus jujuba* cv. Jinsixiaozao, Yazao, Jianzao, Junzao, Sanbianhong (Chinese jujube)	Total sugar (%, *w*/*w*)		80.86–85.63	[15]
	Reducing sugar(%, *w*/*w*)		57.61–77.93	
	Dietary fiber (%, *w*/*w*)		6.13–8.90	
	Lipid (%, *w*/*w*)		0.37–1.02	
	Protein (%, *w*/*w*)		4.75–6.86	
	Moisture (%, *w*/*w*)		17.38–22.52	
	Ash (%, *w*/*w*)		2.26–3.01	
	Potassium (mg/100 g)		79.2–458	
	Phosphorus (mg/100 g)		59.3–105	
	Calcium (mg/100 g)		45.6–118	
	Manganese (mg/100 g)		24.6–51.2	
	Iron (mg/100 g)		4.68–7.90	
	Sodium (mg/100 g)		3.22–7.61	
	Zinc (mg/100 g)		0.35–0.63	
	Copper (mg/100 g)		0.19–0.42	
	Thiamin (mg/100 g)		0.04–0.09	
	Riboflavin (mg/100 g)		0.05–0.09	
	Vitamin C (mg/100 g)		192–359	
	total phenolic content (mg/100 g)		5.18–8.53	
*Z. mauritiana* Lamk. (Indian jujube)	Moisture (%, *w*/*w*)	81–83		[17]
	Carbohydrate (%, *w*/*w*)	17		
	Protein (%, *w*/*w*)	0.8		
	Fiber (%, *w*/*w*)	0.6		
	Lipid (%, *w*/*w*)	0.07		
*Yak jujube* and *Bokjo jujube* (Korean jujube)	Moisture (%, *w*/*w*)	71.46–72.9		[18]
	Protein (%, *w*/*w*)	1.37–1.71		
	Lipid (%, *w*/*w*)	0.31–0.33		
Grande de *Albatera jujube* and *Dátil jujube* (Spanish jujbue)	Moisture (%, *w*/*w*)	78.3–82.1		[12,19]
	Protein (%, *w*/*w*)	0.37–0.61		
	Total soluble solids (% Brix)	17.73–24.07		
	Fiber (%, *w*/*w*)	0.7–1.0		
	Potassium (g/kg)	11.9–17.3		
	Calcium (g/kg)	0.23–0.72		
	Magnesium (g/kg)	0.40–0.77		
	Sodium (g/kg)	0.11–0.43		
	Iron (mg/kg)	10.2–17.1		
	Zinc (mg/kg)	4.0–5.8		
	Copper (mg/kg)	0.5–1.2		
	Manganese (mg/kg)	0.2–2.9		
*Z. jujube* cv. Huizao, Jinzao, Dalongzao, Hupingzao, Popozao, Shenglizao, Zanhuangzao, Guanyangduanzao, Lailutangzao, Lichengxiaozao, Nanjingyazao, Pinglujianzao, Shanxilongzao, Xiangfenyuanzao, Tengzhouchanghongzao (Chinese jujube)	Total flavonoids (mg RE/g FW)	0.47–2.00		[24]
	Total polyphenol (mg GAE/g FW)	0.558–2.520		
	Total triterpene (mg UAE/g FW)	7.52–16.57		
	Total polysacchrides (mg/g FW)	3.103–21.815		
	cAMP (μg/g FW)	17.38–193.93		
	Proanthocyanidins (mg CE/g FW)	0.511–0.977		
	Ascorbic acid (mg/g FW)	1.671–4.247		

Notes: cAMP—cyclic adenosine monophosphate, RE—retinol equivalent, GAE—gallic acid equivalent, UAE—ursolic acid equivalent, CE—catechin equivalent, FW—fresh weight, DW—dry weight.

Among the 5 varieties of jujube grown in the cultivated area of Loess Plateau in China, the total polyphenolic compound content was 428.5–600.4 mg GAE/100 g FW, and the total flavonoid content was 159.3–230.3 mg GAE/100 g FW [25]. In the following year, the team also analyzed 10 varieties of jujube from Loess Plateau for specific analysis, and a total of 11 phenolic compounds were detected: gallic acid, protocatechuic acid, cinnamic acid, chlorogenic acid, caffeic acid, ferulic acid, ellagic acid, catechins, epicatechins, rutin, and quercetin, of which, rutin, catechin, and epicatechin were the major compounds in jujube extracts [14]. In addition to polyphenolic compounds, the content of vitamin C (58.0–413.7 mg/100 g FW) was also positively correlated to the strength of antioxidant capacity [14].

Polysaccharides are formed from multiple monosaccharide molecules, in which the composition and configuration of glycosidic bonds of monosaccharides may lead to the diversity of polysaccharide structure, thus affecting pharmacological activity [26]. Combined with previous studies, jujube polysaccharides are one of the main active components of jujube with the effects of immune regulation and promotion of gastrointestinal activity [27]. Zhao et al. isolated acidic polysaccharides from Jinsixiaozao through ethanol extraction, which consisted of rhamnose, arabinose, galactose, and galacturonic acid, and found this polysaccharide could enhance immune regulatory effects by inducing the proliferation of rat splenocytes in a dose-dependent manner [28]. In addition, Wang et al. extracted jujube polysaccharides by ultrasonic extraction, and the in vitro anti-tumor studies showed that this acidic polysaccharide could inhibit the growth of liver cancer cells and be applied in cancer prevention and treatment [29]. Through different extraction methods, Huizao polysaccharides were obtained, and Zou et al. found that these polysaccharides were mainly acidic pyranose, with a unique glycosidic bond → 4)-α-D-Glcp-(l → or containing galacturonic acid and amide functional groups, which could increase the richness of *Lactobacillus* and *Bifidobacterium bifidum*, thereby exhibiting certain potential for probiotic activity [30]. It can be seen that there is a certain correlation between the extraction methods of jujube polysaccharides and their biological activity.

Triterpenoic acid is mainly in the form of free acids or glycosidic groups of triterpenoid saponins, and has been proven to be one of the most characterized active components of jujube plants [31]. The side-chain structures of triterpenoids are prone to oxidation, reduction, and hydroxylation, so their structural diversities also lead to different pharmacological activities [32]. Pan et al. summarized the structural types of free triterpenoic acids in jujube, which were lupane, oleanane, ursane, and ceanothane configurations, with the ceanothane-type having the highest number of compounds [31]. The research team of Guo used reverse-phase HPLC for the first time to detect dried jujube, and obtained 10 triterpenoid acid compounds, including ceanothic acid, alphitolic acid, zizyberanal acid, zizyberanalic acid, epiceanothic acid, ceanothenic acid, betulinic acid, oleanolic acid, ursonic acid, and zizyberenalic acid [33]. The team also detected the content of triterpenoid acids in jujube from different regions and parts, and found that the content of triterpenoid acids in jujube from the Ningxia region was twice as large as that from the Xinjiang region [34]. Moreover, only three types of triterpenoid acids belonging to the lupane type were found in the kernels and seeds of wild jujube. Therefore, the content of triterpenoid acids in jujube pulp was higher than that in kernels and seeds. Not only that, Peng and Liu compared the content of triterpenoid acids in different varieties and jujube parts and found that the content of betulinic acid and ursolic acid in the leaves of Jinsixiaozao was higher than that in jujube pulp [35]. Thus, the difference in the content of triterpenoid acids depended on the varieties and location of jujube and the growing conditions. In the growth stage of jujube, the total content of triterpenoid acids continuously increased, and reached its highest level in the later stage of fruit ripening (total triterpenoid acid content 6130 ug/g) [36]. Triterpenoids from jujube could induce apoptotic cell death in human cancer cells through mitochondrial reactive oxygen species production. Shin et al. extracted eight triterpenoid acids from jujube, and cytotoxicity assays were performed through using human lung adenocarcinoma cells, prostate carcinoma cells, and breast cancer cells to evaluate the anticancer activity of triterpene acids. The results showed that coumaroyl alphitolic acid isomers of alphitol-type had IC50 values ranging from 8.2 to 14.7 on these cancer cells, and all of them had the effect of reducing the survival rate of cancer cells; additionally, their anticancer activity was found to be stronger than that of betulinic acid through analyzing these apoptotic signaling pathways [37]. Thus, triterpene acids in jujube have potential biological activity in the treatment of some diseases.

Nucleosides are the basic building blocks of DNA, and nucleobases serve as cofactors for enzymes and other proteins. Both play important roles in maintaining physical health and have various biological activities [6,7]. Zhao et al. determined cAMP and cyclic guanosine monophosphate (cGMP) by HPLC and discovered that the content of cAMP and cGMP in jujube peel was the highest, followed by the pulp and leaves [38]. Guo et al. used UPLC-DAD-MS to quickly identify nucleosides and nucleobases in 49 jujubes and found that the total content in Jinzao reached the highest at 1239 µg/g DW [39]. Wang et al. found that the cAMP and cGMP contents of fresh jujube fruits were 130.94 and 150.2 µg/g DW, respectively, and indicated that hot air drying could retain more nucleosides [40]. In recent studies, Hua et al. summarized the nucleoside and nucleobase content in jujubes, in which the higher content of cytidine monophosphate (CMP) was 107.94 µg/g and that of guanylate monophosphate (GMP) was 83.58 µg/g [7]. Studies have indicated that some nucleosides in jujube could be used as an anti-allergy drug for anti-allergy treatment. By injecting crude peanut protein extract into mice to establish a mouse model of peanut allergy, Sang et al. found that allergic symptoms were suppressed in mice treated with jujube cAMP extract. The reduced serum levels of specific immunoglobulin E and the recovery of the spleen index showed that cAMP had a certain anti-allergic effect [41].

## 4. Processed Jujube Products

Fruit processing is an important part of the food industry. Due to the direct decay of fruits after harvesting, processing is an effective method to extend the storage period of fruits. As people pay more attention to nutrition and health, processing technology is also improving ways to retain more original nutrients. 

Meanwhile, proper processing of fruits can also increase flavor characteristics, thereby attracting more consumers to love them. Jujubes are usually sold as simple processed products (such as dried jujubes, jujube powder, etc.). In order to ensure the quality and nutritional value of jujube products, it is necessary to study and summarize the changes in flavor and nutritional content of jujube products of various processing methods. Table 3 summarizes the pros and cons of processing methods for dried jujube and its processed products, providing certain reference value for extending the shelf life of jujubes.

### 4.1. Dried Jujube

**Figure 2:**
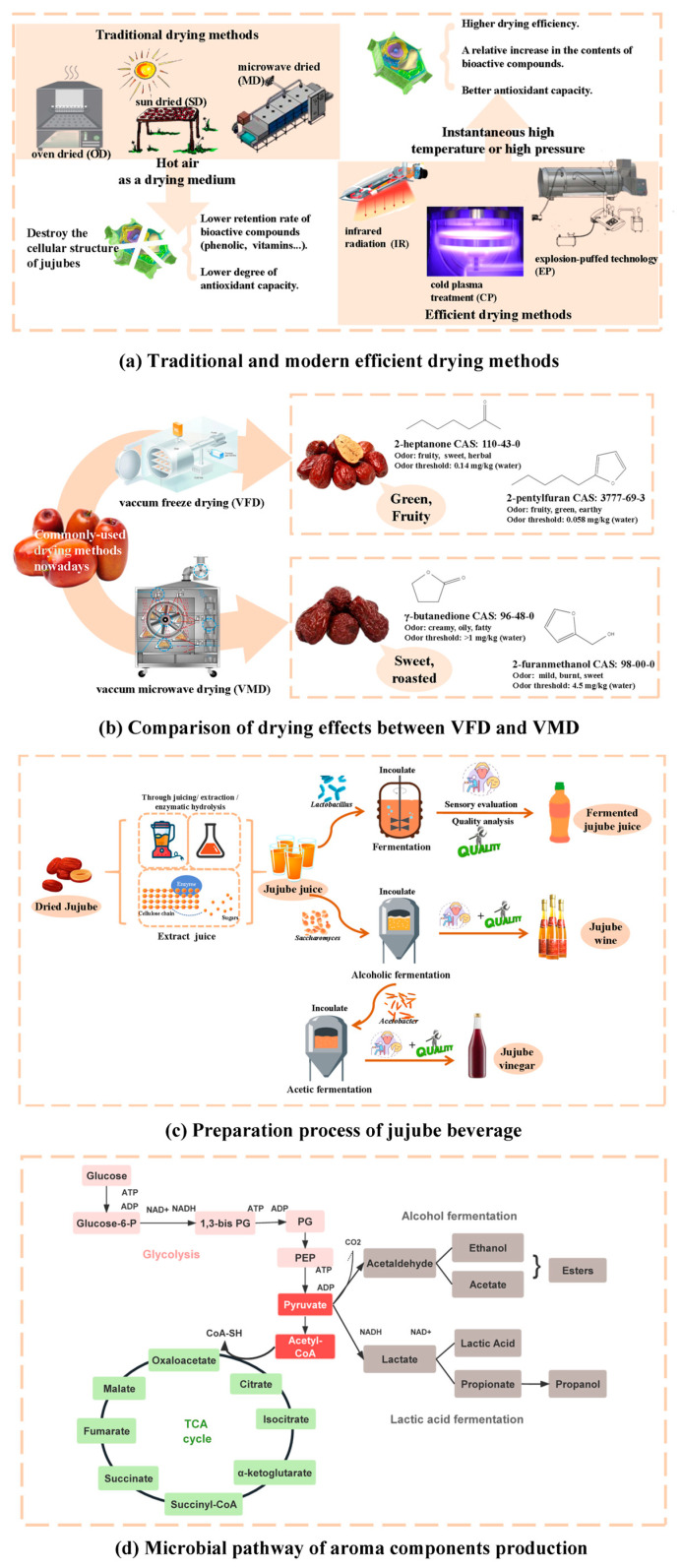
Comparison of drying methods for jujubes, production process of deep-processed products, and main aroma production pathways during deep processing: (**a**) traditional drying methods versus modern efficient drying methods; (**b**) drying effects in VFD and VMD; (**c**) preparation process of jujube beverage; and (**d**) microbial pathway of aroma components production.

The core process of the primary processing of jujube is drying, such as freeze-dried jujube, jujube powder, etc. Although the level of processing is low, the removal of water from jujube makes it easy to be transported and stored. Currently available drying techniques are freeze drying (FD), vacuum drying (VD), hot air drying (HAD), microwave drying (MD), and microwave vacuum freeze drying (MVFD) [42]. These drying techniques have greatly improved the drying efficiency and have ensured the quality of jujubes compared to traditional drying. Figure 2a illustrates traditional and modern efficient drying methods.

The most traditional drying methods include sunlight drying (SD), oven drying (OD), freeze-drying (FD), and microwave drying (MD), of which, SD was found to be better for the retention of carbohydrate content in jujubes, and the content of malic acid significantly increased after OD, while MD increased the content of catechin and epicatechin [14]. However, the ability to scavenge (2,2′-azino-bis(3-ethylbenzothiazoline-6-sulfonic acid) diammonium salt (ABTS+) decreased, which may be attributed to the decrease in antioxidant capacity due to the deactivation of some enzymes under heating conditions. Therefore, FD without heating demonstrated its advantages, as it preserved the cellular structure of the plant well and the total phenolic content increased by 91 mg/kg DW compared to fresh jujubes [14]. Similiar to the principle of HAD was convection drying (CD); Wojdyło et al. studied the effect of CD and VMD on the bioactive components of jujubes. They concluded that the content of vitamin C after VMD was 3036–3270 mg/100 g DW, the flavonoid content ranged from 2937 mg/100 g to 3714 mg/100 g DW, and flavonoid content ranged from 983–1362 mg/100 g DW, so the jujube from CD and VMD had a better antioxidant capacity, with a scavenging rate of 38.1–41.8 mmol Trolox equiv/100 g DW against ABTS radicals [27].

With the rapid development of technology, innovative drying technologies are gradually emerging. Explosion-puffed drying technology (EPD) is a fast and inexpensive drying method that rapidly evaporates the internal moisture. In recent years, it has been used in the drying of jujubes. Du et al. found that the total polyphenolic content of dried jujubes after EP increased by 41.14 mg GAE/g DW compared to fresh jujubes, proving that this drying technology can improve the antioxidant capacity of jujubes [43]. At the same time, instantaneous high-temperature and high-pressure technology is more environmentally friendly and feasible. The wavelength range of short- and medium-wave infrared radiation (SMIR) is 1–4 μm, which brings faster drying capacity and makes it more energy-efficient while reducing the possibility of bioactive substances being sensitive to heat, indicating that SMIR has better prospects in the drying technology of fruits [44]. 

Recently, an efficient pre-treatment method for jujube has gradually begun to replace traditional food preservation methods. In cold plasma treatment (CP), the gas is subjected to high-frequency voltage to produce active particles, generating ionized gas, and causing various physical and chemical changes [45]. When CP was treated around jujubes for 60 s and then HAD was used at 50 °C, the content of total polyphenolics and flavonoids increased to a certain extent compared to direct HAD (by 6.13% and 53.44%, respectively) [46]. It is clear that the pre-treatment method of CP can inhibit the degree of thermal degradation of polyphenolics and flavonoids, thereby retaining the antioxidant capacity of fresh jujube fruits to a greater extent.

In addition, volatile organic components (VOCs) are also one of the indicators for evaluating food quality. The temperature selection and heating time of the drying methods will also have effects on VOCs of jujubes (Figure 2b). For example, through sensory evaluation groups evaluating the color, aroma, and flavor of dried jujubes, it was found that higher temperatures in VMD methods generally increased the bitterness and hardness of jujube peels [28]. While Song et al. found that VFD jujubes had the highest content of acetic acid, propionic acid, and butyric acid, which were acid compounds, the drying method with the largest variety of VOCs was the instant controlled pressure drop drying, which generates a green and fruity aroma such as 1-octen-3-ol, 2-heptanone, and 2-pentyl-furan [47]. Thus, low-temperature or vacuum treatment is considered to be the most suitable drying method for retaining VOCs of jujubes or other fruits. Liu et al. found that HAD at 60 °C could obtain VOCs with larger odor active values (OAVs), such as 2-hexenal, 1-nonanal, and benzaldehyde [48], which were also recognized as fresh and green aroma components by sensory panels. However, as the temperature increased (70 °C and above), some fatty acids were prone to oxygenation to produce furan compounds or Maillard reaction products. Similarly, the study by Yan et al. showed that the longer the drying time, the more negative the effects on the flavor of jujubes. By analyzing the effects of VMD technology on the VOCs in jujubes at different times, it was found that with the extension of drying time, the content of furan compounds with roasted and bitter flavors gradually increased (such as 2-furanomethanol), while the content of roasted aroma compounds with caramel aroma, such as 2,3-butanedione and γ-butanedione, reached its peak at 2.5 min of microwave drying time [49]. From this, it can be concluded that drying technology and the selection of temperature and time will have a certain impact on the composition of aromatic substances, which provides a theoretical basis for improving flavor quality and producing high-quality dried jujubes.

### 4.2. Jujube Beverage

Jujube juice beverage is made by adding sugar and acid to the appropriate amount of raw fruit juice or concentrated fruit juice. The production of jujube juice is a method of deep processing of jujube, which can enhance the economic returns of jujube [50]. However, the water content in the fresh jujube is less, and it needs to be filtered by adding water to pulp in order to obtain jujube juice. Therefore, there have been many studies on the preparation process and quality of jujube beverage. Table 4 focuses on the flavor impact on jujube beverage. Figure 2c,d summarizes the preparation process of jujube beverage and the main microbial pathway of aroma production.

#### 4.2.1. Jujube Juice

Zhao et al. obtained jujube juice by enzymatic hydrolysis and sterilization after juicing jujube with water, and under the stability tests, they observed that ascorbic acids and polyphenolic contents decreased rapidly, and so did aldehydes and ketones, thus leading to a worse flavor [51]. However, different sterilization methods for jujube juice can also have impacts on the nutrition of jujube. Shen et al. used high hydrostatic pressure (HHP) to compare the sterilization effects and showed that the retention rate of ascorbic acid content was the highest with HHP at 600 MPa (342.4 mg/100 g) [52], and the total polyphenolic content in jujube juice only decreased by nearly half, thus also retaining the ability to scavenge DPPH· to some extent.

Most of the jujube juice in previous studies was produced by pulping or juicing with water, but this was less productive and the juice yield was poor. Yu et al. used ultrasonic and microwave extraction methods to extract jujube juice, and discovered that the soluble solids content of jujube juice increased 6.7%, and the solid content to reach the equilibrium time was only 2 h [53]. Thus, the combined methods can improve the yield of fruit juice. Some researchers also suggested that some polysaccharides may hinder the release of soluble solids during juice extraction, thereby reducing the extraction rate of juice. Patel et al. used cellulase enzymatic hydrolysis to optimize juice extraction, and finally obtained the theoretical optimal soluble solid content of jujube juice of 14.20%, total polyphenolics content of 0.94 g GAE/100 mL, and the radical 2,2-diphenyl-1-(2,4,6-trinitrophenyl)hydrazyl (DPPH·)-scavenging rate of 82.41% [54]. Therefore, the enzymatic extraction of jujube juice provides a reliable basis for improving the juice yield of jujubes, and also saves costs and improves efficiency in the preparation of jujube juice beverages.

#### 4.2.2. Fermented Jujube Juice

Microbial fermentation products, due to their complex metabolic pathways and composition of biological enzymes, can improve product flavor and taste and enrich nutritional value. As a non-dairy probiotic food, fermented jujube juice is gradually being accepted and loved by consumers [8]. Park et al. fermented jujube juice with yeast and acetic acid bacteria, respectively, obtaining high antioxidant activity, tyrosinase inhibition rate, and antibacterial activity [55]. Jin et al. screened the mixed starter from the juice of homemade pickled vegetables, with *Lactobacillus casei* and *Lactobacillus rhamnosus* as the dominant bacteria. After fermenting for 7 days, the jujube lactic acid beverage was obtained and it was found that the lactic acid bacteria-fermented beverage showed higher antioxidant capacity than other beverages: the DPPH·-scavenging capacity was 223.8 ± 2.4 mg ascorbic acid (VC) equiv/L and the ABTS·+ scavenging capacity was 1126.64 mg VC equiv/L [56]. This proved that the fermented products of jujube have excellent biological activity and can enhance the additional product value of jujubes. In addition, the reducing sugars rich in jujubes are considered suitable substrates for lactic acid bacteria fermentation. By mixing different *Lactobacillus* bacteria for fermenting jujube juice, the results showed that jujube juice fermented by the mixture of *L. rhamnosus* (LGG) and *L. plantarum* (LP) contained 2.663 mg/mL of total polyphenolics and 0.164 mg/mL of total flavonoids, which were higher than those of other lactic acid bacteria mixtures, and the number of live bacteria increased to 9.15 Log CFU/mL after fermentation [57]. This provided new ideas for the flavor innovation of lactic acid bacteria beverages, and jujubes can be used as potential nutritional components in probiotic foods.

There have also been many studies on the flavor components of fermented jujube juice. Using an electronic nose and an electronic tongue to combine the flavor analysis of three different lactic acid bacteria-fermented jujube juice samples of *L. casei*, *L. plantarum*, and *E. faecium*, Cai et al. found that jujube juice fermented by *L. plantarum* had a weak response to the WW sensors (being sensitive to sulfur-containing compounds) of the electronic nose, while the WC sensors (being sensitive to aromatic compounds) had a higher response than the fermentation juice of the other two lactic acid bacteria, and it had weaker bitterness, astringency, and corresponding aftertaste [58]. This accounted for the high overall acceptance of jujube juice fermented by *L. plantarum*. The inhibitory effect and aroma-producing pathway of *L. plantarum* can be further analyzed in subsequent studies, thus prolonging the shelf-life of fermented jujube juice as well as enriching the flavor and texture of fermented jujube beverages. Pan et al. used three types of lactic acid bacteria to co-ferment jujube juice, and through GC-MS analysis, 7 volatile components contributing significantly to the aroma were identified, such as 1-octen-3-ol, phenyl alcohol, damascone, and some acid compounds [59]. This study laid the foundation for improving the sensory quality of jujube lactic acid bacteria beverage products.

Moreover, in recent years, mixed fermentation has yielded more advantages than co-fermentation of the same type of bacterial strains, which can improve the aroma and flavor of fruits and vegetables. The mixed fermentation of *L. plantarum* and *S. cerevisiae* can not only increase the content of free phenolic acids to improve the antioxidant capacity, but also produce more esters and aldehydes to provide more fruity and floral flavors to the fermented beverages of jujubes [60]. In further study, non-*Saccharomyces* organisms, such as *Candida albicans* and *Pichia pastoris*, can be considered in the selection of mixed fermentation agents in mixed fermented jujube juice.

#### 4.2.3. Jujube Wine

The general fermentation steps of jujube wine are similar to those of common fruit wine fermentation. The sugar content and pH of the fruit are adjusted after pulping pretreatment, and sulfur dioxide and fermentation agents are added for fermentation. Finally, the alcohol concentration and residual sugar content are measured to determine the termination time of fermentation [61]. It has been shown that jujube wine not only had a strong and unique jujube aroma after fermentation, but also retained a certain amount of nutrients [62]. Liu and Zhao determined the following optimal fermentation conditions with active dry yeast for wine: sugar content of 18%, pH value of 4.0, inoculation concentration of 0.3%, and fermentation temperature of 24 °C. Under these optimal conditions, the residual sugar content reached 1.5 g/L and vitamin C content was 350 mg/L, which was in line with the standard of fruit wine, and it had the aroma of fresh jujube, with a sweet and mellow taste [63].

Since the microbial fermentation process and the diversity of outcome products are complex, the components of jujube wine undergo rapid changes during production. Tang et al. analyzed the flavor components of jujube wine through using an e-nose and an e-tongue during three stages of jujube wine fermentation: the early stage of fermentation (0–24 h), the middle stage (36–132 h), and later stage (144–240 h). In the early stage of fermentation, there were many aromatic compounds, while the response values of sulfur-containing organic compounds increased in the middle stage of fermentation, and, in the later stage of fermentation, the acidity, richness, and other taste components of jujube wine significantly increased [64]. This provided a reference and theoretical basis for the dynamic changes in the quality of jujube wine during the fermentation process. The following year, the team analyzed the aroma quality and biological activity of jujube wine and found that the fermentation substrate may affect the quality of jujube wine. For example, fermented jujube juice with jujube peel not only contained more aromatic components than filtering peel, but also enhanced the flavor complexity and aroma intensity of the jujube itself [65]. Moreover, because the jujube peel contained most of the bioactive components in the whole jujube, the total polyphenolic content in jujube juice fermented with jujube peel was about 14 mg/mL, and the DPPH·-scavenging activity was greater than 95% [65]. From this, it can be concluded that fermentation with jujube peel could maximize the retention of bioactive substances in jujube and enhance their flavor complexity. Not only that, Cai et al. found that different pre-treatment methods (direct pulping, pectinase hydrolysis, etc.) may also affect the VOCs of jujube wine, especially alcohols and acetate esters, such as 3-methylbutanol, phenyl alcohol, ethyl caproate, ethyl decanoate, etc., and the optimal pretreatment method was pectinase enzymatic hydrolysis followed by fermentation [66].

Although fruit wine brewing has been conducted for hundreds of years, with the changes in consumer tastes and the increasing competition from other low-alcohol wines, jujube wine also needs continuous innovation and product quality improvement to meet market demands. The fermentation agent of fruit wine is mainly *S. cerevisiae*, and the aroma production pathways of different yeasts also affect the aroma composition of fermented jujube wine. Zhao et al. compared the VOCs of 6 *S. cerevisiae* fermented jujube wines and concluded that there were some differences in the composition of VOCs by different brewer’s yeasts, and the total ester content of jujube wine fermented by *S. cerevisiae* BV818 was the highest, at 91.82 mg/L (such as ethyl hexanoate, with pineapple aroma, and phenyl alcohol, with rose aroma), and that with the lowest ester content was *S. cerevisiae* RMS2. Similarly, the sum of the scores of each sensory description in the sensory evaluation was also the highest for BV818 [67]. Zhang et al. suggested that co-fermentation was a valuable method to enhance jujube wine flavor because they added both lactic acid bacteria and *P. pastoris* to *S. cerevisiae,* which resulted in higher overall acceptance of the final jujube wine than that of the single *S. cerevisiae* fermentation and had a more mellow flavor and less bitterness [68]. The conclusion of this study improves the sensory characteristics and acceptability of jujube wine, but the ratio of the inoculated mixed bacteria needs to be further investigated to provide a reference for the research and development of the mixed fermentation agents suitable for the fermented jujube wine.

#### 4.2.4. Jujube Vinegar

Vinegar is generally produced by inoculating vinegar with a special starter or acetic acid bacteria for fermentation. The production of jujube vinegar was first made from jujube wine through yeast fermentation for 10–20 days, and then the liquid was transferred to another container, inoculated with acetic acid bacteria, and fermented at 30 ± 2 °C for about 20 days so as to obtain jujube vinegar [69]. Jujube vinegar is rich in a variety of organic acids, such as gallic acid, chlorogenic acid, ferulic acid, p-coumaric acid, and other bioactive components, such as polyphenolics and melanoids, and has many benefits for human health, like antibacterial, anti-inflammatory, antioxidant, and anti-diabetes effects. Since there is a possibility of cracking or premature dropping of fresh jujubes during the ripening period, Xiang et al. used these defective jujubes for processing and fermentation to obtain vinegar and found that the organic acid composition of the defective jujube vinegar was similar to that of vinegar processed from generally harvested jujube, mainly consisting of acetic acid, malic acid, and lactic acid [70]. This method allows for the large-scale and rapid production of jujube vinegar products from defective jujubes, which not only saves a lot of energy but also reduces the waste of jujube resources.

Wang et al. directly added edible alcohol to adjust the alcohol concentration of jujube juice to around 6%, and inoculated it with *Acetobacter aceti* for fermentation. Through response surface optimization experiments, the optimal fermentation conditions for black jujube vinegar were determined to be 15% *A. aceti* inoculation, 30.44 °C fermentation time, and initial alcohol content of 6.45% vol. The theoretical total sugar content of vinegar after fermentation was 21.52 ± 0.23 mg/mL, with a cAMP content of 6.60 ± 0.04 μg/mL. The ability to scavenge ABTS·+ was 0.52 ± 0.03 mg Trolox/mL [71], indicating that jujube vinegar had the ability to retain or produce high levels of nutrients after fermentation. Similarly, Buadak fermented jujube juice through two fermentation stages of ethanol and acetic acid, resulting in a soluble solid content of 4.75%, titratable acid content of 6.35 g/100 mL, total polyphenolics content of 982.83 mg GAE/L, and an antioxidant capacity of 11.49 mmol Trolox/L [72]. It can be seen that jujube vinegar is a product with high nutritional value, and jujube vinegar with high antioxidant and total phenolic content was prepared by fermentation of fresh jujubes.

Some researchers held that jujube vinegar accumulated the most flavor substances in the middle and later stages of fermentation. The highest content in jujube vinegar was undoubtedly acetic acid, while the compounds that contribute more to the flavor were acetate esters, such as ethyl acetate, phenylethyl acetate, isoamyl acetate, 2-methylbutyl acetate, etc. It can be seen that acids and esters have been identified as the main compound categories of jujube vinegar [72]. What is more, Ruan et al. analyzed VOCs and amino acid substances. The results showed that 6 bitter amino acids and 5 sweet amino acids could soften the overall sourness of jujube vinegar and promote its overall acidity [73]. The formation of volatile flavor compounds, such as ethyl benzoate and ethyl hexadecanoate, with a fruity aroma appeared in the post-fermentation stage, giving jujube vinegar a unique aromatic aroma.

**Table 3:** Pros and cons of different processing methods of jujube.

Processing Method		Advantages	Disadvantages	Reference
Drying	Explosion-puffed drying (EPD)	Instantaneous high-temperature and high-pressure technology.	Need pre-treatment to remove a certain amount of moisture	[6]
	Sun drying (SD)	Better retention rates in fructose and surcose.	High requirements for weather	[14]
	Oven drying (OD)	Increased the main organic acid content in jujubes.	Heat was transferred from the outside to the inside, need long duration	
	Microwave drying (MD)	Better retention rate of phenolic compounds.	Led to enzyme inactivation	
	Freezing drying (FD)	No need for heating and preserves the cellular structure in the fruit.	Slow dehydration speed and high energy consuming	
	Short- and medium-wave infrared radiation drying (SMIRD)	Faster and more efficient than convective heating.	Infrared radiation heated too quickly	[20]
	Vacuum microwave drying (VMD)	Higher retention rate of active ingredients.	Easier to dry unevenly	[27]
	Cold plasma treatment	Quickly improved drying efficiency, and increased the content of total polyphenols and flavonoids.	Suitable for drying small materials	[46]
	Convenience level of drying process: VMD/VFD > SMIRD, EPD > MD/FD > OD > SD.			
Juice	Jujube juice with high hydrostatic pressure (HHP)	Non-heating process, reducing color browning, retaining more flavor and nutrients.	Prone to loss of phenolics, leading to a decrease in antioxidant capacity	[52]
	Jujube juice using ultrasound and microwave extraction method	Increase in soluble solids content and increased juice yield.	Produced more foam or sediment	[53]
	Jujube juice through cellulase enzymatic hydrolysis method	Decomposed the polysaccharide components in jujubes.	Diverse sources of enzymes, may have inadaptability	[54]
	Enzymatic hydrolysis improves juice production efficiency and HHP has better sterilization effect.			
Fermented products	Jujube juice fermented by yeast and acetic acid bacteria	High antioxidant activity and high antibacterial activity.	Need to study the adaptability of bacterial strains to substrates in advance, and the fermentation process was difficult to control	[55]
	Jujube juice fermented by mixed lactic acid bacteria fermented jujube juice	Much higher antioxidant capacity.		[56]
	Jujube juice fermented by mixed lactic acid bacteria fermented jujube juice	The number of live probiotics was greater than 10^8^ CFU/mL.		[57]
	Jujube wine fermented by commercial *Saccharomyces cerevisiae*	Jujube pulp and peel fermented together had more aromatic components and also enhanced the complexity and intensity of the flavor of jujube itself.		[58]
	Jujube vinegar fermented by *Acetobacter*	More flavor substances accumulation.		[73]
	Better biological activity and more volatile aroma components than jujube juice.			

### 4.3. Innovative Jujube Products

Nowadays, with people’s increasing attention to food health and dietary therapies that can prevent diseases, jujube is experiencing an increasing demand in the consumer market. The various nutrients and functional substances rich in jujubes can also be applied to many innovative food products in order to obtain functional foods.

Yuan et al. selected three common foods: pumpkin, mango, and jujubes, to obtain a mixed beverage of fruits and vegetables. Through GC-MS, hexanal and (E)-2-octanal were considered to be the most important compounds in jujube, followed by hexanoic acid and octanoic acid. The content of these volatiles also played a significant role in the evaluation of the sensory group, giving the composite juice a sweet floral, green, and fresh taste, as well as a unique acidity of the fruit, with good overall acceptance [74]. Not only that, bamboos are also a high-yield ingredient in China, with a delicious taste and high nutritional value. Zhao et al. first inoculated and fermented a composite beverage obtained by co-extracting bamboos and jujubes. The total polyphenolic content was 32.92 mg/L, the total flavonoid content was 95.81 mg/L, the sucrose content decreased by 44.1% after fermentation, and the scavenging rate of hydroxyl radicals increased by 5.788% [75]. At the same time, the main components were ketones, alcohols, and acids, indicating that fermentation can reduce the sugar content in high-sugar fruits and enrich the flavor of composite fruit drinks.

**Table 4:** Flavor enhancement from different microbial fermentation agents.

Fermentation Strain	Key Findings about Flavor Characteristics	Research Significance	Reference
*Lactobacillus plantarum*	E-nose: higher response values of WC sensors	Extending the shelf-life of fermented jujube juice and enriching the flavor.	[58]
	E-tongue: weak bitterness, astringency, and their corresponding aftertaste.		
*L. plantarum*, *L. rhamnosus GG*, and *Streptococcus thermophilus*	GC-MS-O: phenylethyl alcohol, E-2-octenal, 1-octen-3-ol, trans-damascenone, benzoic acid methyl ester.	Revealing the aroma production mechanism of lactic acid bacteria.	[59]
*S. cerevisiae* BV818	GC-MS: 3-methylbutanol, phenylethyl alcohol, octanoic acid ethyl ester, decanoic acid ethyl ester.	Enzymatic hydrolysis gives highest content of volatile aroma components.	[66]
*L. plantarum*, and *Saccharomyces cerevisiae*	LC-MS: Higher efficiency of carbohydrate degradation, acid production, and ethanol-production.	Enhancing the quality of fermented jujube juice and enriching the flavor and taste of jujube juice.	[68]
	GC-IMS: octanoic acid ethyl ester, hexanoic acid ethyl ester, 2,3-pentanedione, cyclohexen-2-one.		
Jujube vinegar fermented by *Acetobacter*	HPLC: acetic acid, malic acid, citric acid, lactic acid, aspartic acid, glutamic acid, arginine.	The amino acids produced by acetic acid bacteria can soften the overall acidity of jujube vinegar and promote the formation of ethyl volatile flavor compounds.	[73]
	GC-MS: ethyl phenylacetate, phenylethanol.		
Different commercial *S. cerevisiae*	GC-MS: hexanoic acid ethyl ester, acetic acid phenylethyl ester, pentanol, butanol.	The ratio of ester compounds produced by *S. cerevisiae* to the content of isoamyl alcohol has significant impacts on the overall flavor of jujube wine.	[75]

In addition to making composite drinks through juice extraction, jujube extract can also retain certain nutritional components and biological activity. Gharavi et al. obtained jujube extract and added a certain proportion of jujube extract to the cake formula for dough fermentation and baking. The results showed that the cake with 6% jujube extract had a certain improvement in hardness after baking compared to the 4% addition, and the sensory score of the cake with 6% added was higher than that of the other added amounts and the control group [76]. Further research can be conducted on the physical and chemical properties of jujube cake. Similarly, regarding baked products, researchers have proposed using jujube powder as a partial substitute for wheat flour to make pizza dough. It was found that the total polyphenolic content and antioxidant capacity of the dough increased with the increase in the proportion of jujube powder in the dough (when the amount of jujube powder addition was 7.5%, the total phenolic content was 1.51 mg GAE/g DW, the total flavonoid content was 0.11 mg QE/g DW, and the ABTS·+ scavenging rate was 78.52%). Moreover, the baked dough had higher hardness, viscosity, and elasticity, making it more chewy and nutritious [77]. From this, it can be concluded that compared to single-wheat flour, both jujube extract and jujube powder have excellent biological activity and high dietary fiber content, providing new ideas for the production of dough and even other functional baking products.

## 5. Pharmacological Effects of Jujube and Its Products

The bioactive component content and pharmacological effects of jujube and its products were significant. As a medicinal and food homolog, these bioactive components played a significant role. This section investigated the extensive health-promoting effects of jujube and jujube-based foods. Table 5 summarizes the pharmacological effects of jujube and its processed products.

### 5.1. Antioxidant Activity

Phenolic acid compounds are naturally found in various plants, including jujube, and are basically composed of phenolic acids, flavonoids, and tannins. These compounds have strong antioxidant capacity and can fight free radicals and other reactive oxygen species to promote human health. Many studies have proven that jujube peel is the best antioxidant part of the whole fruit, with strong antioxidant capacity. Xue et al. discovered that the DPPH-scavenging rate of all kinds of jujube peels was 1.5–1.8 times that of pulps, and the FRAP value of jujube peel was 75% to 85% of the positive control [78]. However, some studies have reported that the antioxidant properties of phenolic acids and their derivatives were related to the position, number, and substitution sites of hydroxyl groups on the aromatic ring, mainly divided into free phenolic acids, esterified phenolic acids, glycosided phenolic acids, and insoluble-bound phenolic acids. Wang et al. isolated and identified 8 phenolic acids in different parts of jujube, including hydroxybenzoic acid, hydroxycinnamic acid, and chlorogenic acid. The results showed that there were more phenolic acids in the form of glycosides in jujube pulp, and insoluble-bond phenolic acids in jujube peel and seed. And jujube peel had very high antioxidant activity, the DPPH free radical-scavenging rate was higher than 80%, and the FRAP value was between 600 μmol and 680 μmol FeSO4/g DW [79]. This suggests that the glycosided and insoluble-bond phenolic acids in jujube may help to scavenge free radicals or disrupt the chain reaction that terminates free radicals.

Ascorbic acid is a water-soluble compound that mainly scavenges free radicals by inhibiting the chain reaction of free radicals [12,80]. The content of bioactive components and antioxidant activity of 15 jujubes were analyzed and determined by Kou et al. It showed that the content of ascorbic acid ranged from 1.671 mg/g to 4.247 mg/g FW, and the free radical-scavenging rate of ABTS+ was 0.959–1.195 mM TE/100 g FW. FRAP values were 224.62–406.18 mg VC equiv/100 g FW [24]. Owing to the variety of resources and genetic diversity of jujube, the content of ascorbic acid in Spanish varieties was 387–555 mg/100 g FW, the free radical scavenging rate of ABTS·+ was 28.85–43.73 mM Trolox equiv/100 g DW, and the FRAP value was 17.6–34.82 mM Trolox equiv/100 g DW [27]. Both of these studies indicate that ascorbic acid has a stronger correlation with ABTS+ and FRAP, proving that ascorbic acid is another major component of jujubes that plays an antioxidant role.

However, the phenolic compounds and ascorbic acid content decreased with the growth stage of jujube. Jujube at the young fruit stage (ascorbic acid content of 851–1120 mg/100 g FW, total phenolics content of 26.19–35.06 mg GAE/g DW) was suitable for natural antioxidants, while in the later ripening stage, due to the continuous increase in the content of fructose and glucose, it was more suitable for sale as a fresh fruit or a processed food [81]. To avoid the decline in nutritional quality during storage after harvesting, Yao et al. found a potential antisepsis treatment method through methionine treatment to increase the content of total polyphenolics and flavonoids. After storage, the content of total polyphenolics increased by 35 mg GAE/100 g FW after methionine treatment, and more free flavonoids were generated during storage. The scavenging rate of ABTS+ and DPPH· showed a slowly decreasing trend [82]. In conclusion, methionine treatment can improve the antioxidant capacity of jujubes, and phenolic substances and flavonoid content have a greater impact on the antioxidant activity of ABTS·+ and DPPH·.

Fermented jujube products have higher biological activities compared to single jujube extracts. Ren et al. compared the chemical components of jujube extract and jujube fermented wine for the first time using UPLC-HR-MS. In the DPPH- and ABTS+- scavenging experiments, the IC50 values of jujube extract were 32.48 mg/mL and 91.52 mg/mL, respectively, while the IC50 values of jujube wine were 11.05 and 13.96 mg/mL, respectively [83]. In addition, by studying the effects of in vitro digestion on the antioxidant activity of jujube vinegar, Li et al. found that although gastric digestion reduced the content of total polyphenolics (reduced by 54.17%), gastric protease and gastric acid could maintain a high clearance rate of DPPH·. In intestinal digestion, the total polyphenolic content increased by about 5–9%, and the scavenging rate of DPPH· was also improved to a certain extent (23.86% in the first 0.5 h) [84]. This study provided a theoretical basis for the stable functionality of jujube vinegar during digestion, and also proved the high antioxidant activity of jujube vinegar.

### 5.2. Anticancer Activity

Traditional Chinese medicine for the treatment of cancer has attracted more and more attention, and jujube extracts are gradually being used to treat or alleviate various diseases. Plastina et al. obtained three kinds of jujube extracts by *n*-hexane, chloroform, and ethyl acetate extraction, and studied the effect of the extracts on breast cancer cells. Estrogen receptor alpha (ERα)-positive MCF-7 cells and ERα-negative SKBR3 breast cancer cells were determined by 3-(4,5-dimethylthiazol-2-yl)-2,5-diphenyltetrazolium (MTT) assay to analyze the vitality of cell proliferation, and through immunoblotting analysis and DNA fragment gap staining, apoptosis was analyzed. The results showed that the IC50 values of the three jujube extracts on ERα-positive MCF-7 cells were 14.42, 7.64, and 1.69 μg/mL, respectively, and on Erα-negative cancer cells, were 14.06, 6.21, and 3.70 μg/mL, respectively [85]. Thus, jujube extracted with ethyl acetate had a greater proliferation reaction and played an anti-breast cancer agent function. By pretreating jujube to obtain jujube deproteinized polysaccharides, Huang et al. evaluated the anti-proliferative effect of jujube polysaccharides on melanoma cells cultured in Eagle’s medium using MTT assay. The results showed that the survival rates of melanoma cells reached 45.46% and 18.19% after 24 h of cultivation with two doses of 4.25 mg/mL and 5 mg/mL of jujube polysaccharides. Fluorescence microscopy observation and cell cycle analysis confirmed that melanoma cells eventually stagnated in the G2/M phase, leading to abnormal growth and division [86]. Therefore, it was concluded that deproteinized jujube polysaccharides can be used as a drug for treating skin cancer.

The jujube extract was also obtained by solvent extraction, and the cytotoxic effect of jujube extract in vitro was evaluated by the MTT method. Hoshyar et al. added a certain concentration of jujube extract during the culturing of the OV-2008 line of cervical cancer cells, and used the IC50 value of jujube to calculate cell survival rate. They found that the IC50 values of jujube extract at 24, 48, and 72 h were 1.2, 0.5, and 0.2 mg/mL, respectively, and jujube extract upregulated the expression of P53, P21, and P27 and downregulated the expression of cyclin D1 in cervical cancer cells, thereby limiting the proliferation of cervical cancer cells and reducing the toxicity of cancer cells [87]. Moreover, recent studies have shown that jujube extract can also disrupt the homeostasis of cancer cells, causing cancer cell number reduction. Hepatocellular carcinoma (HepG2) was cultured with different concentrations of jujube phenolic extracts. Shi et al. calculated that the IC50 value of jujube was in the range of 0.1–0.8 mg/mL, and the HepG2 was negatively correlated with the concentration of jujube extracts, with a correlation coefficient of 0.9349 [88]. These results indicate that jujube extracts can inhibit the proliferation of liver cancer.

### 5.3. Anti-Obesity Activity

Obesity is considered a risk factor associated with the occurrence or development of a variety of diseases, and also a more serious health problem. Kubota et al. studied the effect of jujube extract on the enzyme activities of 3 T3-L1 adipocytes and glycerol-3-phosphate dehydrogenase (GPDH). The results indicated that jujube extract obtained by chloroform extraction could better inhibit intracellular triglyceride accumulation and GPDH activity in 3 T3-L1 adipocytes, and the addition of jujube extract could reduce the expression of some adipocyte transcription factor-producing proteins [89]. For example, adipogenic transcription factors PPARγ, C/EBPα, and C/EBPβ can block the generation of fat to achieve an anti-obesity effect.

Since jujube powder was the most accessible product after dried jujube, Deshpande et al. studied the effect of jujube powder on obese mice fed a high-fat diet for 90 days, and divided the mice into five groups: normal mice, obese mice, obese mice treated with 250 mg/kg jujube powder daily, obese mice treated with 500 mg/kg jujube powder daily, and obese mice treated with 0.9 mg/kg sibutramine daily. The weight of obese mice in the 250 mg/kg jujube powder group decreased by 16.33% and fat mass decreased by 68.99%, while the weight of the 500 mg/kg jujube powder group decreased by 17.38% and fat mass decreased by 72.84%, and the weight of obese mice fed with medicine only decreased by 5.52% [90]. Therefore, the anti-obesity effect of jujube powder is significantly higher than that of sibutramine.

### 5.4. Anti-Diabetes Activity

Diabetes is closely related to insulin, a hormone secreted by the pancreas that regulates blood glucose balance, but type 2 diabetes can occur when there is insufficient insulin. Animal studies have shown that large amounts of sugar intake can reduce insulin sensitivity [91]. Zhao et al. conducted intragastric experiments on mice and divided them into the following groups: normal saline, 20% high-fructose water with 0 mg/kg, 200 mg/kg, or 400 mg/kg jujube polysaccharides, and drinking water with 200 mg/kg or 400 mg/kg jujube polysaccharides. The results showed that serum insulin concentration increased by 1.65 times in the mice fed only high-fructose water. Compared with the mice fed only fructose water, the insulin concentration decreased by 12.5% and 38.4%, respectively, and almost did not affect the glucose and insulin levels of the saline control group mice [67]. It can be seen that the higher the molar ratio of insulin to glucose, the greater the degree of insulin resistance.

In addition, diabetes is characterized by hyperglycemia and abnormal carbohydrate, lipid, and protein metabolism, and in the metabolic pathway, serum adiponectin is an important hormone regulating glucose and fatty acid metabolism. By studying the effect of jujube water extract on serum adiponectin levels in diabetic mice, Hemmati et al. concluded that after two weeks, mice with diabetes treated with jujube extract had significantly decreased the levels of serum triglycerides and VLDLs (86.7% and 86.5%, respectively) compared with the control group. The blood glucose levels were even lower than those of healthy mice [92]. Subsequently, Yazdanpanah’s team randomly selected 116 patients with type 2 diabetes and divided them into two groups: a balanced diet with three meals, and water infused with jujube powder before three meals for controlled human clinical trials. After 12 weeks of trials, the results showed that glycosylated hemoglobin (HbA1c) decreased by 0.68%, total cholesterol decreased by 24.29 mg/dL, and triglycerides decreased by 43.3 mg/dL in patients taking jujube powder [93]. It can be seen that jujube powder can significantly improve the glycosylated hemoglobin and blood lipid levels of patients with type 2 diabetes, so as to achieve the effect of treatment and prevention of type 2 diabetes.

Nevertheless, because the content of fructose and glucose in jujube juice is higher than 350 g/L, jujube juice was first enzymatically hydrolyzed by D-glucose isomerase and D-allose-3-epimerase, and then fermented with mixed lactic acid bacteria. It was found that a portion of fructose and glucose were converted into low-calorie and sweet D-allolose, which accounts for 15% of all reducing sugars in fermented jujube juice. And fermentation accumulation produced many branched-chain amino acids, such as Val, and Leu, which help promote insulin release and increase the nutritional value of the fermented jujube juice [94]. Moreover, jujube products are rich in dietary fiber, which is usually not easily digested by digestive enzymes, and can increase insulin sensitivity by blocking the absorption of glucose, thereby preventing diabetes [95].

### 5.5. Improvement of Immunity

Using jujube and its extracts instead of antibiotics is a new method in aquaculture. The bioactive components of jujube may stimulate immune-related cytokines in fish skin, thereby reducing disease mortality in aquaculture. Through continuously feeding common carp (*Cyprinus carpio*) juveniles a diet containing jujube extract for 8 weeks, there was no significant difference in the lysozyme activity in the skin mucus between the 0.5% jujube extract group and the common carp group. The total immunoglobulin level was significantly higher than that of the normal juvenile group (increased by 75%). In addition, the expression of cytokines (interleukin 1β, interleukin 18, and tumor necrosis factor-α) in juvenile skin was also significantly up-regulated in the 0.5% jujube extract group [96]. These cytokines play an important role in skin inflammation and immune response, and the up-regulation of their expression indicates the improvement in autoimmune ability. Thus, it is proven that jujube extract has a potential effect on improving the immune properties or immune parameters of carp skin mucosa.

Zhuang et al. fed cyclophosphamide (CTX) and jujube powder to mice inoculated with colon tumor cells and observed the changes in tumor cells. The experiment discovered that the jujube powder and cyclophosphamide stimulated the enrichment of CD8+T cells in cyclophosphamide, which not only helped to stimulate white blood cells in peripheral blood and bone marrow, thereby inhibiting the growth of eosinophils in peripheral blood and restoring T lymphocytes, but also increased the diversity of gut microbiota without causing an imbalance [97]. More propionate and butyrate were produced to improve immunity and inhibit the growth of colon tumors. The team then looked at the gut microbiota of the mice and the response to vaccination to reveal whether there was a vaccine immune effect between the two. The results found that when mice were injected with the antibiotic mixture and fed with jujube powder at the same time, they could increase the macrophage B cells in mesenteric lymph nodes, memory B cells, and plasma cells in peripheral blood, and significantly increase the concentration of specific immunoglobulin lgG1 in serum. Furthermore, jujube powder improved the production of some amino acid metabolites, which increased the abundance of intestinal microbiota, thereby activating the intestinal immune response of mice to the vaccine [98].

**Table 5:** Study on pharmacological activities of jujube and its products.

Pharmacological Activities	Study Object	Jujube Samples	Main Observation	Reference
Antioxidant activity	2,2′-azinobis (3-ethylbenzothiazoline-6-sulfonicacid) (ABTS+),ferric-reducing antioxidant power (FRAP)	15 varieties of Chinese jujube	The correlation coefficient of the content of bioactive compounds and antioxidant activity was R^2^ ABTS·+ = 0.659, R^2^ FRAP = 0.668.	[24]
	ABTS+, FRAP	3 varieties of Spanish jujube	The values of ABTS·+ and FRAP were 28.85–43.73 mM TE/100 g DM, 17.6–34.82 mM TE/100 g DM, and the correlation coefficient between asorbic acid and ABTS·+ or FRAP was higher than 0.6.	[27]
	2,2-diphenyl-1-(2,4,6-trinitrophenyl)hydrazyl(DPPH), FRAP	Jujube peels and pulps in 3 varieties	The DPPH-scavenging rate of jujube peels was 1.5–1.8-fold higher than in pulps and the FRAP value was 75% to 85% of the positive control.	[78]
	DPPH, FRAP	3 different parts of jujube (peel, pulp and seed)	Glycosided and insoluble-bond phenolic acids had higher antioxidant activity, which mainly presented in jujube peel, having 46.23 mg GAE/gDW content of total phenolic compounds.	[79]
	DPPH, ABTS+	Jujube extract and jujube fermented wine	The IC50 values of jujube fermented wine were 11.05 and 13.96 mg/mL, much lower than that of jujube extract.	[83]
	In vitro gastrointestinal digestion	Green jujube vinegar	Jujube vinegar could secrete more gastric protease and gastric acid during digestion, and the scavenging rate of DPPH· was improved by 23.86% in the first 0.5 h.	[84]
Anticancer activity	Estrogen receptor alpha (ERα) positive MCF-7 cell, ERα-negative SKBR3 breast cancer cell	Jujube extracts with different solvents (*n*-hexane, chloroform, and ethyl acetate)	The IC50 values of the 3 jujube extracts on ERα positive MCF-7 cells were 14.42, 7.64, and 1.69 μg/mL, and 14.06, 6.21, and 3.70 μg/mL on Erα-negative cancer cells. The ethyl acetate extract of jujube had apoptosis effect on breast cancer cells.	[85]
	Skin melanoma cells	Jujube deproteinized polysaccharides	After 48 h of cultivation, the survival rates of cancer cells decreased to only 15.46% and 13.36% with two doses of this jujube polysaccharides, 4.25 mg/mL and 5 mg/mL, respectively.	[86]
	OV-2008 line cervical cancer cells	Jujube extracts with distilled water	The IC50 values of jujube extract at 24, 48, and 72 h were 1.2, 0.5, and 0.2 mg/mL, respectively, and jujube extract upregulated the expression of P53 and P21 and downregulated the expression of cyclin D1 in cervical cancer cells.	[87]
	Hepatocellular carcinoma (HepG2)	Jujube phenolic extracts of different concentration (0.05–1.00 mg/mL)	The IC50 value of jujube extract was in the range of 0.1–0.8 mg/mL, and the correlation coefficient between hepatocellular carcinoma and jujube extracts was 0.9349.	[88]
Antiobesity activity	3 T3-L1 adipocytes, Glycerol-3-phosphate dehydrogenase (GPDH)	Commercial jujube extracts	Jujube extract could better inhibit intracellular triglyceride accumulation and GPDH activity in 3 T3-L1 adipocytes, and reduce the expression of some adipocyte transcription factor-producing proteins.	[89]
	Obese mice fed with high-fat diet	Jujube powder	The weight of obese mice in 250 mg/kg jujube powder group decreased by 16.33% and fat mass decreased by 68.99%, while the weight of 500 mg/kg jujube powder group decreased by 17.38% and fat mass decreased by 72.84%.	[90]
Anti-diabetes activity	Mice fed with high fructose water	Jujube polysaccharides	Mice fed with high fructose water and 400 mg/kg jujube polysaccharides could decrease blood glucose concentration by 10.0%, and decrease insulin concentration by 38.4%.	[67]
	Mice with diabete	Jujube extracts	Mice fed with jujube extract showed significantly reduced serum triglyceride and VLDL levels, with 86.7% and 86.5% reductions, respectively.	[92]
	Patients with type 2 diabetes	Jujube powder	The glycosylated hemoglobin (HbA1c) decreased by 0.68%, total cholesterol decreased by 24.29 mg/dL, and triglyceride decreased by 43.3 mg/dL in patients taking jujube powder.	[93]
Immunity improvement	Skin mucus of *Cyprinus carpio* juveniles	Jujube extracts	Diet with 0.5% jujube extracts could significantly raise skin mucinase activity and total immunoglobulin level. And the expression of cytokines in juvenile skin was also significantly up-regulated.	[96]
	Cyclophosphamide (CTX)	Jujube powder	Jujube powder stimulated the enrichment of CD8+T cells in cyclophosphamide as well as white blood cells in peripheral blood and bone marrow.	[97]
	Diversity of gut microbiota of mice with HSA vaccination	Jujube powder	Injection with the antibiotic mixture and feeding with jujube powder could increase the macrophage B cells in mesenteric lymph nodes and significantly increase the concentration of specific immunoglobulin lgG1 in serum.	[98]

## 6. Conclusions and Future Prospects

Jujubes are a homolog of medicinal and edible food with rich nutritional value and bioactive components for the human body, such as essential amino acids, polysaccharides, polyphenolics, and triterpenoid acids. However, due to the short shelf-life of ripe jujubes after harvesting, simple or deep processing of jujubes can increase the additional value of its products and better meet the increasing demand of consumers for healthy food. The existing research has confirmed that the dried and powdered jujube obtained by the most simple processing method and fermented products, such as jujube wine and jujube vinegar, all have good antioxidant properties, anti-cancer activity, anti-obesity activity, and anti-diabetes and immunity-enhancing effects. Therefore, jujubes have high application potential and may be developed into edible products with multiple health functions. For example, adding jujube polysaccharides and triterpenoid acids as nutritional supplements to jujube powder or some jujube-flavored foods can endow nutritional foods with the effects of regulating probiotic activity and improving immunity; for instance, by utilizing the polyphenolic compounds and various vitamins rich in jujubes, combined with medicinal herbs, such as *Tremella fuciformis* and Chinese wolfberries, innovative beauty products and skincare foods can be developed to attract consumers of different age groups to purchase such products.

However, processed jujube products still face some challenges, so several future prospects are proposed: (1) different strains of fermentation are used, and further exploration is needed to explore the metabolic pathways between aroma-producing microorganisms and aroma components, as well as the correlation between microbial metabolites and volatile components; (2) studies on bioactive components in jujube products are currently based on animal experiments, and it is necessary to conduct human clinical trials to better verify the bioactive components of jujube and understand their pharmacological mechanisms; (3) deeply processed jujube products need to be commercialized in order to be more well-known to consumers and solve the problems of unsold dried jujube fruits and waste of jujube resources; (4) the development of innovative jujube functional food relies on flavor instrument analysis, sensory analysis, bioactivity assay, and safety toxicology research. Future research needs to combine these methods to bring consumers higher quality nutritional products.

## Figures and Tables

**Table 1:** Examples of planting areas and climatic characteristics of different varieties of Chinese jujubes resources.

Planting Area	Cultivation Area	Climate Characteristics	Characteristic Varieties of Jujube	10 Representative Jujube Varieties in China
North of the Qinling-Huaihe River	Cultivation areas in the middle and lower reaches of the Yellow River, Haihe River, and Liaohe River Basin	Annual sunshine hours: 2300–2900 h. Average annual temperature: 7–14.5 °C. Average annual rainfall: from July to August, with a precipitation of 500–800 mm. The growth period of jujube trees: about 200 d.	*Ziziphus jujuba* cv. Jinsixiaozao, *Ziziphus jujuba* cv. Linyilizao, *Ziziphus jujuba* cv. Goutouzao	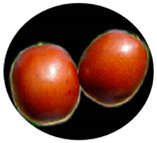 Jingsixiaozao 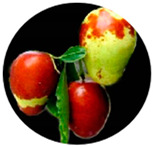 Lizao 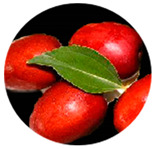 Goutouzao 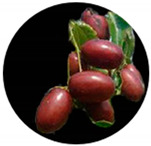 Hetianyuzao 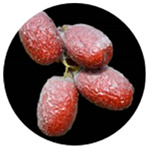 Huizao
	Cultivated areas on the Loess Plateau	Annual sunshine hours: 2200–2800 h. Average annual temperature: 8.5–12 °C. Average annual rainfall: from July to September, with a precipitation of 400–600 mm. The growth period of jujube trees: about 175–195 d.	*Z. jujuba* cv. Hetianyuzao, *Z. jujuba* cv. Muzao, *Z. jujuba* cv. Youzao, *Z. jujuba* cv. Dongzao	
	Valley cultivation area in the northwest arid region	Annual sunshine hours: 2600–3250 h. Average annual temperature: 6.5–10 °C. Average annual rainfall: little rain and is always drought. The growth period of jujube trees: about 160–180 d.	*Z. jujuba* cv. Zanxindazao, *Z. jujuba* cv. Ruoqianghuizao, *Z. jujuba* cv. Hetianyuzao, *Z. jujuba* cv. Lingwuchangzao	
South of the Qinling-Huaihe River	Cultivation area of alluvial soil in the Jianghuai River	The climate is variable: rainy in spring and early summer, with high temperatures and short-term drought in summer, so the flowering periods are affected by rainy seasons. The growth period of jujube trees: about 200–225 d.	*Z. jujuba* cv. Yiwudazao, *Z. jujuba* cv. Sihongdazao, *Z. jujuba* cv. Xuanchenyuanzao	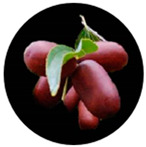 Muzao 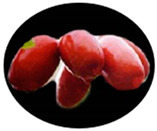 Youzao 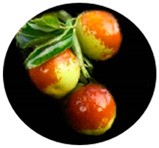 Dongzao 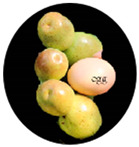 Yiwudazao 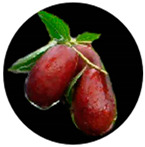 Guanyangchangzao
	Cultivation area of southern tropical and subtropical regions	Annual sunshine hours: 1600–2100 h. Average annual temperature: 18–24 °C. Average annual rainfall: with a precipitation of 1200–1800 mm. The growth period of jujube trees: about 240–290 d. Especially, the fruit yield is relatively high, and harvested twice a year.	*Z. jujuba* cv. Lianxianmuzao	
	Cultivation area of Yunnan, Guizhou, and the western Sichuan Plateau	Annual sunshine hours: 1200–2400 h. Average annual temperature: 13–17 °C. Average annual rainfall: with a precipitation of 200–1200 mm. The growth period of jujube trees: not detailed, from May to October.	*Z. jujuba* cv. Mudongxiaotianzao, *Z. jujuba* cv. Guanyangchangzao	

## Data Availability

No new data were created or analyzed in this study. Data sharing is not applicable to this article.
